# Mice deficient in the C-terminal domain of TAR DNA-binding protein 43 develop age-dependent motor dysfunction associated with impaired Notch1−Akt signaling pathway

**DOI:** 10.1186/s40478-019-0776-5

**Published:** 2019-07-25

**Authors:** Kohei Nishino, Seiji Watanabe, Jin Shijie, Yuri Murata, Kotaro Oiwa, Okiru Komine, Fumito Endo, Hitomi Tsuiji, Manabu Abe, Kenji Sakimura, Amit Mishra, Koji Yamanaka

**Affiliations:** 1 Department of Neuroscience and Pathobiology, Research Institute of Environmental Medicine, Nagoya University, Chikusa-ku, Nagoya, Aichi 464-8601 Japan; 2 Department of Neuroscience and Pathobiology, Graduate School of Medicine, Nagoya University, Nagoya, Aichi 466-8550 Japan; 3 Department of Biomedical Science, Graduate School of Pharmaceutical Sciences, Nagoya City University, Nagoya, Aichi 467-8603 Japan; 4 Department of Animal Model Development, Brain Research Institute, Niigata University, Niigata, 951-8585 Japan; 5 Cellular and Molecular Neurobiology Unit, Indian Institute of Technology Jodhpur, Jodhpur, Rajasthan 342011 India

**Keywords:** Amyotrophic lateral sclerosis (ALS), TAR DNA-binding protein 43 (TDP-43), Motor dysfunction, TDP-43 knock-in mice, Notch1, Akt

## Abstract

Intracellular mislocalization of TAR DNA-binding protein 43 (TDP-43), a nuclear DNA/RNA-binding protein involved in RNA metabolism, is a pathological hallmark of amyotrophic lateral sclerosis (ALS). Although the aggregation-prone, TDP-43 C-terminal domain is widely considered as a key component of TDP-43 pathology in ALS, recent studies including ours suggest that TDP-43 N-terminal fragments (TDP-∆C) may also contribute to the motor dysfunction in ALS. However, the specific pathological functions of TDP-43 N-terminal fragments in mice have not been elucidated. Here, we established TDP-∆C knock-in mice missing a part of exon 6 of murine *Tardbp* gene, which encodes the C-terminal region of TDP-43. Homozygous TDP-∆C mice showed embryonic lethality, indicating that the N-terminal domain of TDP-43 alone is not sufficient for normal development. In contrast, heterozygous TDP-∆C mice developed normally but exhibited age-dependent mild motor dysfunction with a loss of C-boutons, large cholinergic synaptic terminals on spinal α-motor neurons. TDP-∆C protein broadly perturbed gene expression in the spinal cords of aged heterozygous TDP-∆C mice, including downregulation of *Notch1* mRNA. Moreover, the level of *Notch1* mRNA was suppressed both by TDP-43 depletion and TDP-∆C expression in Neuro2a cells. Decreased *Notch1* mRNA expression in aged TDP-∆C mice was associated with the age-dependent motor dysfunction and loss of Akt surviving signal. Our findings indicate that the N-terminal region of TDP-43 derived from TDP-∆C induces the age-dependent motor dysfunction associated with impaired Notch1-Akt axis in mice.

**Electronic supplementary material:**

The online version of this article (10.1186/s40478-019-0776-5) contains supplementary material, which is available to authorized users.

## Introduction

Amyotrophic lateral sclerosis (ALS) is an adult-onset neurodegenerative disease characterized by a progressive loss of upper and lower motor neurons in the spinal cord, brain stem, and cerebral cortex, leading ultimately to fatal skeletal muscle paralysis [37]. Aberrant accumulation of TAR DNA-binding protein 43 (TDP-43) is a pathological hallmark of ALS and frontotemporal lobar degeneration (FTLD) [2, 24]. TDP-43 contains a canonical nuclear localization signal, and thus is predominantly localized in nuclei. However, in ALS patients, TDP-43 is partially or completely escaped from the nuclei of motor neurons and glial cells, where it forms cytoplasmic inclusion bodies. Therefore, there may be the combined pathomechanisms of a loss of nuclear TDP-43 function and a gain of cytosolic TDP-43 mediated toxicities [33].

TDP-43 is a ubiquitously expressed DNA/RNA-binding protein shuttling between nucleus and cytoplasm, and plays a critical role in multiple aspects of RNA metabolism such as splicing, stability, transport, translation, and microRNA maturation [5, 27]. TDP-43 has two RNA binding motifs (RRM1 and RRM2, respectively) in its amino (N)-terminal domain (a.a. 1–273). TDP-43 also possesses a prion-like domain with a glutamine and asparagine (Q/N) rich region in the carboxyl (C)-terminus (a.a. 274–414) that confers susceptibility to form aggregates [36]. Missense mutations in the gene encoding human TDP-43, *TARDBP*, have been identified in familial and sporadic ALS, suggesting that TDP-43 dysfunction leads to motor neuron degeneration [16, 33, 37].

Most known ALS-linked *TDP-43* mutations are located in the C-terminal domain [16, 37]. In addition, cleaved TDP-43 C-terminal fragments are accumulated in the lesion of ALS patients [2, 24, 35], and indeed are core components of TDP-43 cytoplasmic inclusions and aggregates [11, 25, 35]. Moreover, we previously reported that aberration of the C-terminal domain disrupted spliceosomal integrity [34]. Therefore, the C-terminal domain of TDP-43 is tightly associated with the ALS pathology. In addition to the C-terminal fragments, the N-terminal fragments of TDP-43 have also been found in the postmortem spinal cord of ALS patients [46]. In the cited study, the N-terminal fragments were produced by the action of calpain, reduced solubility, and sequestered full-length TDP-43 into cytoplasmic aggregates. Intriguingly, another study reported that the alternatively spliced endogenous TDP-43 S6 short variant devoid of the C-terminal domain formed highly insoluble cytoplasmic and nuclear inclusions reminiscent of TDP-43 pathology in ALS [31]. These results suggest that TDP-43 N-terminal fragments may also be involved in TDP-43 pathology. However, the precise pathological mechanisms of TDP-43 N-terminal fragments still remains to be elucidated.

To examine the role of N-terminal TDP-43 fragments in vivo, we established TDP-ΔC knock-in mice (TDP-∆C mice), in which *Tardbp* gene region encoding the C-terminal domain (a part of exon6) is eliminated. Heterozygous TDP-ΔC mice exhibited mild age-dependent motor dysfunction with a loss of C-boutons, large cholinergic synaptic terminals on motor neurons, and suppression of Notch1 − Akt signaling. Suppression of *Notch1* mRNA was induced both by TDP-43 depletion and TDP-∆C expression. Collectively, these results suggest that N-terminal fragments of TDP-43 also contribute to ALS pathology associated with impaired Notch1-Akt signaling pathway.

## Materials and methods

### Animals

Murine *Tardbp* genomic DNA was isolated from C57BL/6 N mouse. The gene targeting vector was designed to replace a part of its exon 6, encoding amino acid 274–414 of murine TDP-43, with 3 × FLAG tag to delete C-terminal portion of TDP-43. We used the genomic fragment spanning from exon 2 to intron 5 and the fragment of 3′-UTR of exon 6 (both the arms are approximately 6 kb) for constructing targeting vectors, respectively. A neomycin resistant gene (*neo*^*r*^) gene cassette with FRT sequences and diphtheria toxin (DTA) cassette were also inserted for positive and negative selection, respectively. The gene targeting was performed by using embryonic stem (ES) cells derived from C57BL/6 N (RENKA) as described elsewhere [19]. The correctly targeted ES cells were microinjected into the blastocysts to generate chimeric mice. The mice heterozygous for TDP-ΔC mutant allele were maintained in C57BL/6 J genetic background, and genotyped by PCR using following primers: 5′-GGCAAACAGCAGTTCACTTTCACCC-3′, 5′-GCTGCTGCTGACTACAAAGACC-3′, and 5′-AGATTTGGTGGTAATCCAGGTGGC-3′. The mice were housed in the specific pathogen free (SPF) environment (12 h light-dark-cycle; 23 ± 2 °C; 50 ± 10% humidity), and treated in compliance with the requirements of the Animal Care and Use Committee, Nagoya University.

### Rotarod test

Rotarod tests were performed as previously reported [44]. In brief, the mice were placed on the rotating rods, which accelerated from 0 to 30 rpm for 5 min with 15 min interval among each trial (Muromachi Kikai, Tokyo Japan). The longest latencies to fall off the rotating rods out of three trials were scored. No randomization or blinding was used in this study.

### Antibodies

Following primary antibodies were used in this study: anti-choline acetyl transferase (ChAT) (1:100, #AB144P, Merck Millipore Corp., Billerica, MA, USA), anti-Kv2.1(K89/34) (1:100, #75–014, RRID: AB_10673392, Neuromab, USA), anti-TDP-43(3H8) (1:2,000 for immunofluorescence, 1:1,000 for immunoblotting, #MABN45, Merck Millipore) anti-TDP-43(A260) (1:1,000, #3449, RRID: AB_2200511, Cell Signaling Technology, Danvers, MA), anti-FLAG M2 (1:5,000, #F1804, RRID: AB_262044, Sigma-Aldrich Co LLC, St. Louis, MO, USA), anti-glial fibrillary acidic protein (GFAP) (1:250, #G3893, RRID: AB_477010, Sigma-Aldrich), anti-Iba1 (1:500, #019–10741, Wako Pure Chemical Industries Ltd., Osaka, Japan), anti-fibrillarin (1:1,000, #2639, RRID: AB_2278087, Cell Signaling), anti-heat shock protein 110 (Hsp110) (1:1,000, #SPA-1101E, RRID: AB_916878, Enzo Life Sciences Inc., Farmingdale, NY), anti-phospho Akt (Ser473) (1:1,000, #4060, RRID: AB_2315049, Cell Signaling), anti-pan Akt (1:1,000, #4691, RRID: AB_915783, Cell Signaling), anti-β-actin (1:5,000, #A5441, RRID: AB_476744, Sigma-Aldrich). Anti-TDP-43 (N-terminal) was raised against a chemically synthesized N-terminal peptide of human TDP-43 a.a. 1–10 conjugated to keyhole limpet hemocyanin (KLH), and rabbit sera following the immunization was used at 1:200 for immunoblotting.

### Immunofluorescence

Immunofluorescence analyses were performed as described elsewhere [43, 44]. Briefly, mice at indicated age were deeply anesthetized, and transcardially perfused with phosphate buffered saline (PBS) then 4%(w/v) paraformaldehyde in 0.1 M phosphate buffer for 10 min, respectively. After the incubation with 30%(w/v) sucrose in PBS, the dissected lumbar spinal cords were embedded in Tissue-Tek OCT compound medium (Sakura Finetek, Tokyo, Japan), and frozen at − 80 °C until use. After blocking, the 12 μm-sliced spinal cord sections were incubated with primary antibodies for overnight at 4 °C. Bound primary antibodies were detected with Alexa Fluor 488-conjugated anti-mouse or Alexa Fluor 546-conjugated anti-goat IgG secondary antibodies (both used in 1:1000) (Thermo Fisher Scientific Inc., Waltham, MA, USA). Immunofluorescence images were obtained by a confocal laser scanning microscopy (LSM-700; Carl Zeiss AG, Oberkochen, Germany) and the equipped software (Zen; Carl Zeiss AG). Cholinergic large synaptic terminals on α-motor neurons (C-boutons) were identified as contacting sites of ChAT and Kv2.1 on the surface of ChAT-positive motor neuron soma in ventral lumber spinal cords. For quantification, more than 50 motor neurons in three animals per genotype were counted for C-boutons based on the immunofluorescence images obtained by confocal laser scanning microscopy.

### Plasmids, cell culture, and transfection

The full-length or ΔC (a.a. 1–273) human TDP-43 cDNA was inserted into pEGFP-N1 vector (Takara Bio, Shiga, Japan) using seamless ligation cloning extract (SLiCE) [20, 51] from *Escherichia coli* HST02 (Takara Bio) to express with C-terminal EGFP tag. Site-directed mutagenesis on TDP-43 cDNA was performed according to the instruction of QuikChange site-directed mutagenesis kit (Stratagene, La Jolla, CA, USA). Mouse neuroblastoma Neuro2a (RRID: CVCL_0470) cells were maintained in Dulbecco’s Modified Eagle’s’ Medium (DMEM) containing 4.5 g/L glucose supplemented with 10%(v/v) fetal bovine serum (FBS), 100 U/mL penicillin, and 100 μg/mL streptomycin (all from Thermo Fisher) at 37 °C in a humidified chamber containing 5% CO_2_. The cells were differentiated in DMEM supplemented with 2%(v/v) FBS and 2 mM N6,2′-O-dibutyryladenosine-3′,5′- cyclic monophosphate (Nacalai Tesque, Kyoto, Japan) for indicated times. Transfection was performed using Lipofectamine 2000 reagent according to the manufacturer’s instruction (Thermo Fisher).

### RNA isolation and quantitative reverse transcription (RT)-PCR

Total RNA of mouse spinal cords was isolated with Trizol reagent (Ambion, Austin, TX, USA), followed by further purification using RNeasy Mini Kit (Qiagen, Hilden, Germany) according to the manufacturer’s instruction. The concentration of total RNA was determined by a spectrophotometer (NanoDrop ND-2000; Thermo Fisher), and RNA quality was assessed with the RNA integrity determined by microfluidics-based capillary electrophoresis (RNA integrity number (RIN) ≧ 8.0) (Bioanalyzer 2100; Agilent Technologies, Palo Alto, CA, USA). cDNA was synthesized from 1 μg of the purified RNA using PrimeScript II 1st strand Synthesis Kit (Takara Bio) and an oligo-(dT)_15_ primer. Quantitative reverse transcription (RT)-PCR was performed using SYBR Premix Ex Taq II (Takara Bio) according to the manufacturer’s protocol in Thermal Cycler Dice Real Time System II (Takara Bio). Relative mRNA expression was calculated by standard curve method normalized to β-actin gene (*Actb*) and relative to the control samples. All samples were run in duplicate. The primers that were used in this study are listed as follows:

for specific detection of mRNA levels of endogenous wild-type TDP-43; 5′-AAAAGGAAAATGGATGAGACAGATG-3′ and 5′-AACTGAGCAGGATCTGAAAGACTATTT-3′, for quantifying mRNA levels of both TDP-ΔC and endogenous wild-type TDP-43; 5′-ATGATAAGGTTGCCCAGTC-3′ and 5′-TACTGTTACCAAACCCACC-3′, for *Notch1*; 5′-TGGATGACCTAGGCAAGTC-3′ and 5′-TTCTGCATGTCCTTGTTGG-3′, for *Hes1*; 5′-TGCCAGCTGATATAATGGAG-3′ and 5′-CTTTGATGACTTTCTGTGCTC-3′, for *Pten*; 5′-AAGGGAGTCACAATTCCCA-3′ and 5′-ACTGAGGATTGCAAGTTCC-3′, for quantifying mRNA levels of β-actin; 5′-GCTATGTTGCTCTAGACTTCG-3′ and 5′-GGATTCCATACCCAAGAAGG-3′.

### Subcellular fractionation

Tissues were fractionated as previously reported [4] with slight modifications. A frozen tissue was homogenized in ice-cold homogenization buffer (10 mM HEPES, 250 mM sucrose, 0.4%(v/v) phenylmethylsulfonyl fluoride (PMSF), pH 7.4) supplemented with protease inhibitor cocktail (Roche Diagnostics, Basel, Switzerland) using Potter-Elvehjem homogenizer (Wheaton Industries, Millville, NJ, USA). The homogenate was centrifuged at 600×g, 4 °C for 5 min. The supernatant was centrifuged at 10,000×g, 4 °C for 30 min, and discard the pellet. The supernatant was further centrifuged at 18,000×g, 4 °C for 30 min, and the resultant supernatant was collected as a cytosolic fraction. The pellet of the first centrifugation step (600×g) was resuspended in ice-cold hypotonic buffer (10 mM HEPES, 10 mM KCl, 1 mM MgCl_2_, 0.5 mM dithiothreitol (DTT), 0.4%(v/v) PMSF, pH 7.4), and incubate for 15 min on ice. After centrifugation at 600×g, 4 °C for 5 min, the pellet was resuspended in ice-cold hypertonic buffer (10 mM HEPES, 400 mM NaCl, 1 mM MgCl_2_, 0.2 mM ethylene glycol tetraacetic acid (EGTA), 30%(v/v) glycerol, 0.5 mM DTT, 0.4%(v/v) PMSF, pH 7.4), and incubated for 30 min at 4 °C with gentle agitation to induce osmotic shock. After centrifugation at 18,000×g, 4 °C for 30 min, the resultant supernatant was collected as a nuclear fraction.

### Immunoblotting

Tissues from control or TDP-∆C mice were sonicated in ice-cold lysis buffer (50 mM Tris-HCl, 150 mM NaCl, 1 mM ethylenediaminetetraacetic acid (EDTA), 1%(v/v) Triton X-100) supplemented with protease inhibitor cocktail (Roche). The lysates were centrifuged at 15,000×g, 4 °C for 5 min to remove insoluble debris. Total protein concentration was measured using Bio-rad protein assay kit as described in manufacturer’s instructions (Bio-rad, Hercules, CA, USA). Aliquots of 20 μg proteins were analyzed by sodium dodecyl sulfate- polyacrylamide electrophoresis (SDS-PAGE), and transferred to a polyvinylidene difluoride membrane (Immobilon-P, Merck Millipore). After blocking with 2% Bovine Serum Albumin (BSA) in TBS-T (50 mM Tris-HCl, 150 mM NaCl, 0.5%(v/v) Tween-20, pH 7.4), the membrane was incubated with the primary antibodies diluted in TBS (50 mM Tris-HCl, 150 mM NaCl, pH 7.4), followed by incubation with horseradish peroxidase (HRP)-conjugated anti-rabbit (1:5,000, #NA934, RRID: AB_772206, GE Healthcare, Waukesha, WI, USA) or anti-mouse (1:5,000, #NA931, RRID: AB_772210, GE Healthcare) secondary antibodies. The membranes were visualized with Immobilon Crescendo Western HRP substrate (#WBLUR0100, Merck Millipore) according to the manufacturer’s protocol. Densitometric analyses were performed by using an image analyzer LAS-4000 mini (Fuji-film, Tokyo, Japan) with the equipped software (Multi Gauge; Fuji-film).

### Microarray analysis

Microarray analyses were performed using purified total RNA of the 700 days-old TDP-∆C mouse spinal cords or their littermate non-transgenic controls. cRNAs were prepared using Low Input Quick-Amp Labeling Kit (Agilent) according to the manufacturer’s instruction, and were hybridized with mouse SurePrint G3 mouse GE microarray 8 × 60 K Ver.2.0 chips (Agilent). Data were subsequently normalized and analyzed using GeneSpring 13.0 software (Agilent). Pathway analyses were conducted using the Single Experiment Pathway analysis feature in GeneSpring 13.0 (Agilent). The moderated t-test was utilized, and 1.2-fold cut-off value was used to select significantly changed transcripts.

### Statistics

Time-course of rotarod score was analyzed by two-way ANOVA and Mann-Whitney non-parametric u-test. All the data from immunofluorescence, semi-quantitative immunoblotting and quantitative RT-PCR were analyzed by an unpaired *t*-test, for comparison between 2 groups, or one-way ANOVA followed by the *post-hoc* Tukey’s multiple comparison *t*-test, for comparison among more than 3 groups, respectively. All the statistical analyzes were carried out using GraphPad Prism software (GraphPad Software, La Jolla, CA).

## Results

### Generation of the mice deficient in the TDP-43 C-terminal domain by gene targeting

**Fig. 1:**
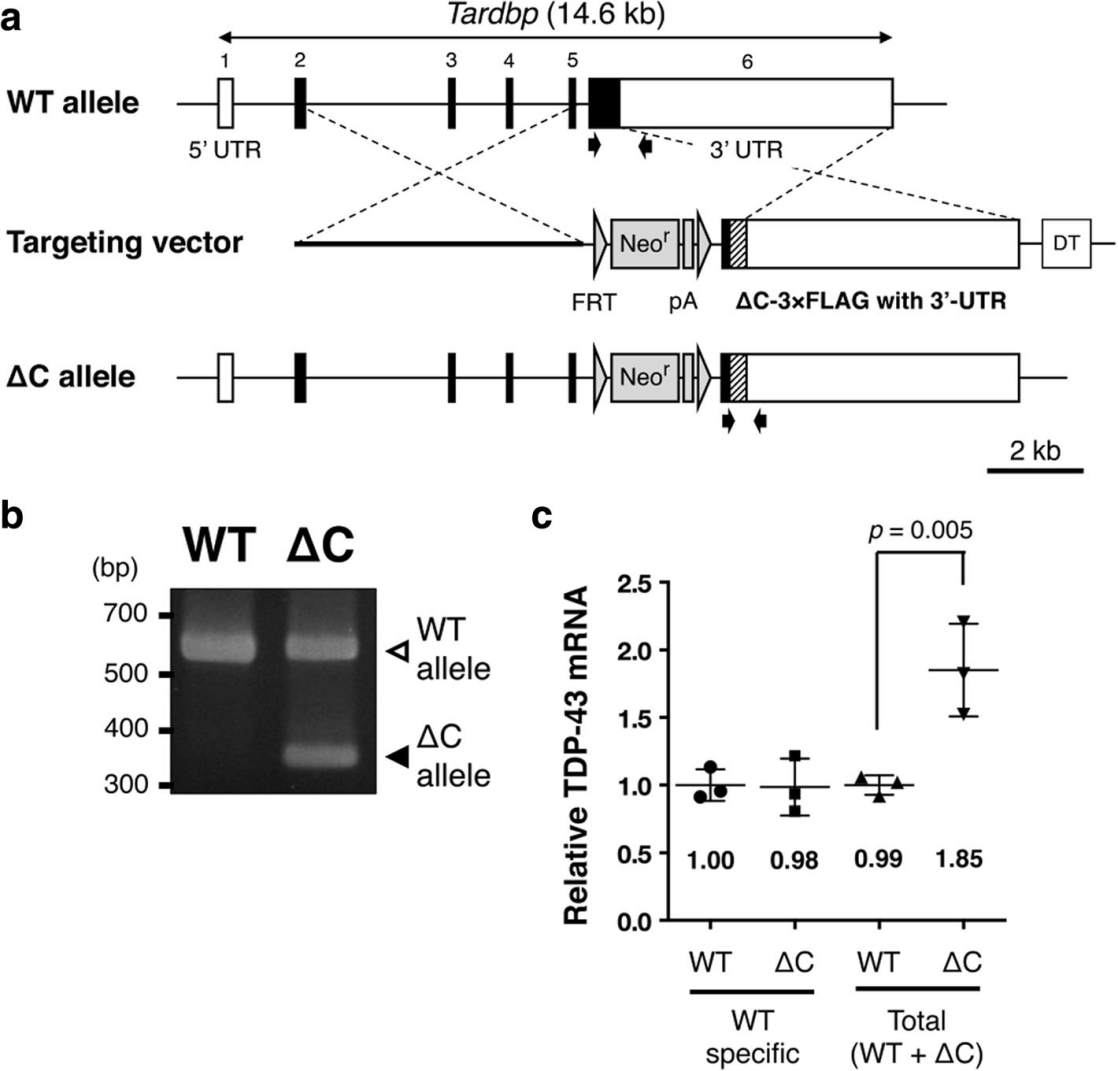
Generation of TDP-∆C knock-in mouse. **a** Schematic diagrams of the murine *Tardbp* gene locus, the gene targeting vector for TDP-∆C knock-in, and the resulting TDP-∆C allele after homologous recombination. A region encoding the C-terminal domain of murine TDP-43 (a.a. 274–414) in *Tardbp* exon 6 was replaced with a 3 × FLAG tag coding sequence. A neomycin resistance gene (Neo^r^) flanked by FRT sequences inserted into the intron 5 and a diphtheria toxin (DT) cassette inserted downstream of intron 6 were used for positive and negative selection, respectively. 3′-UTR, which is crucial for autoregulation of TDP-43 mRNA, remained intact. **b** Representative image for genotyping of wild-type (WT) and heterogeneous TDP-∆C knock-in (∆C) mice. Specific primers used for PCR are indicated by arrows in (**a**). **c** mRNA levels of TDP-43 and TDP-∆C in the spinal cord (SC) of WT and ∆C mice. Quantitative reverse transcription PCR (RT-PCR) was performed with “WT specific” primers, recognizing only endogenous TDP-43 (TDP-WT) cDNA and “total” primers, recognizing both TDP-WT and TDP-∆C cDNAs. Relative mean of TDP-43 mRNA levels normalized to the WT control are plotted with standard deviation (SD). The level of TDP-WT mRNA did not differ between WT and ∆C mice, therefore, the expression level of TDP-∆C mRNA was almost the same as the endogenous TDP-43 mRNA

The previous studies identified various N-terminal TDP-43 fragments with different length of the remaining C-terminal domain produced by the calpain-dependent cleavage [46, 47]. Among the TDP-43 mutants devoid of various length of C-terminal domains, we found that complete deletion of the C-terminal domain from TDP-43 produced most severe neurotoxicity in cultured neuronal cells (Additional file 1: Figure S1). Therefore, to examine pathogenic roles of TDP-43 N-terminal fragments in vivo, we generated the mice completely deficient in the TDP-43 C-terminal domain by gene targeting. A part of exon 6 encoding the murine TDP-43 C-terminal domain (a.a. 274–414) was deleted (Fig. 1a). Mice heterozygous for the TDP-∆C mutant allele were successfully generated (Fig. 1b). When we crossbred the heterozygous mice (TDP-∆C mice) to produce offspring, the ratio of the number of born heterozygous mice to the one of wild-type was roughly 2:1 (heterozygous: 21, wild-type: 9), however no homozygous mice were born, indicating an embryonic lethality of TDP-∆C homozygous mouse as observed in TDP-43 deficient mice. In contrast, TDP-∆C mice, heterozygous for a TDP-∆C allele, were developed normally. The level of endogenous wild-type TDP-43 mRNA was not altered in the spinal cords of TDP-ΔC mice compared to the one in the wild-type (WT) controls (Fig. 1c), likely due to TDP-43 autoregulation mediated by the intact 3′-UTR. On the other hand, the total expression level of endogenous TDP-43 plus TDP-ΔC mRNAs in TDP-ΔC mice was almost twice as high as TDP-43 mRNA expression in WTs (Fig. 1c), indicating that TDP-ΔC mRNA expressed at levels similar to endogenous TDP-43 mRNA in TDP-ΔC mice.

### TDP-ΔC protein is enriched in cytosol, and less stable than the wild-type TDP-43

**Fig. 2:**
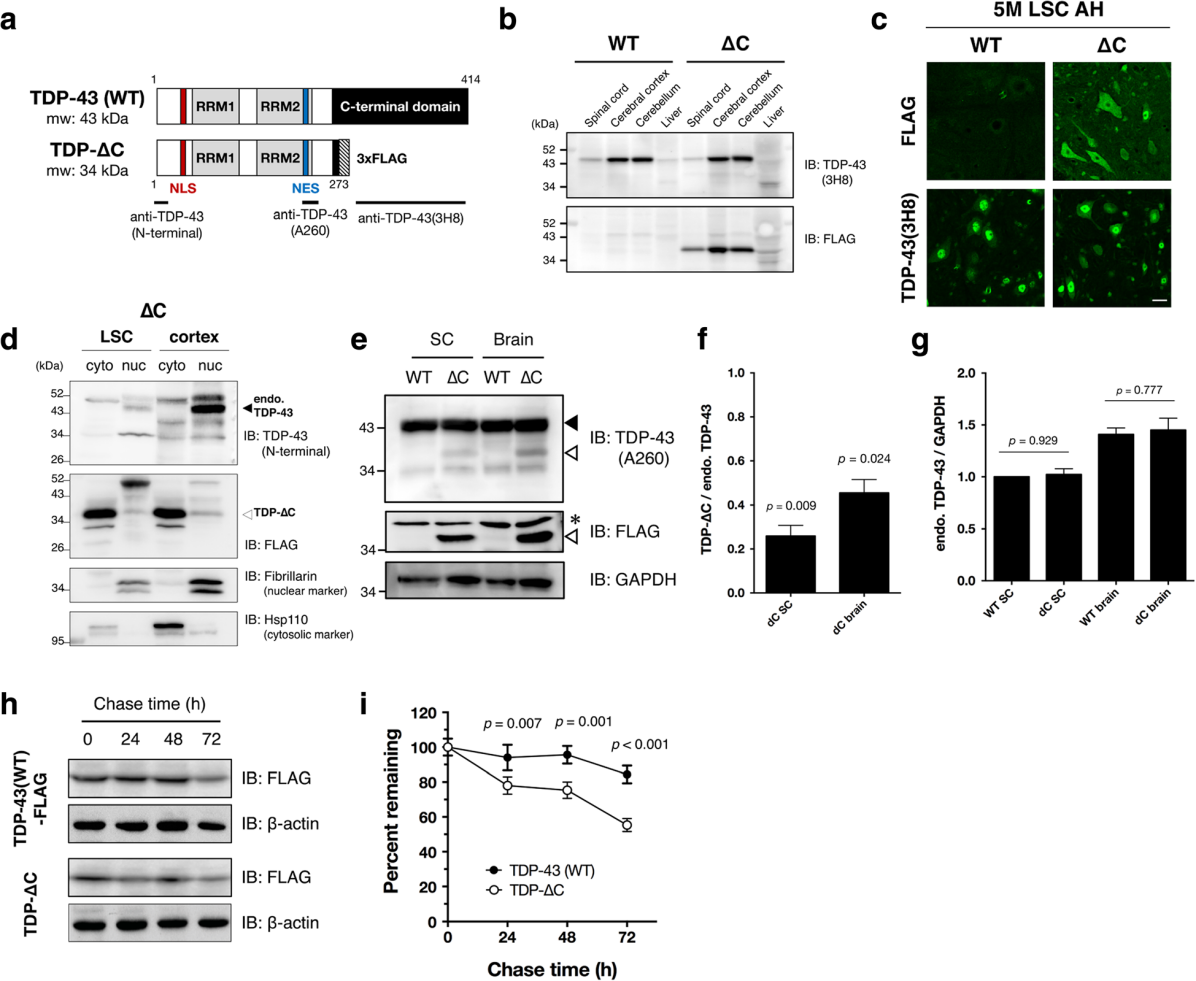
TDP-∆C protein is predominantly localized in cytoplasm, and is less stable than TDP-43. **a** Schematic diagrams of wild-type TDP-43 (TDP-WT) and TDP-∆C proteins. The N-terminal domain of TDP-43 (a.a. 1–273), including the nuclear localization signal (NLS) and the nuclear export signal (NES), remains intact in TDP-∆C protein. Anti-TDP-43(N-terminal) antibody was raised against the extreme N-terminal domain (a.a. 1–10), whereas anti-TDP-43(A260) and anti-TDP-43(3H8) recognizes the region harboring a.a. 260 and the C-terminal domain (a.a. 274–414), respectively. Validation results for these antibodies are shown in Additional file 1: Figure S1. **b** Expression levels of TDP-WT and TDP-∆C proteins in spinal cord, cerebral cortex, cerebellum, and liver of wild-type (WT) and TDP-∆C (∆C) mice. Immunoblotting analyses using anti-TDP-43(3H8) antibody, that specifically recognizes TDP-WT, and anti-FLAG, specific to TDP-∆C, in the indicated tissues of 5-month-old WT and ∆C mice. **c** Representative immunofluorescent images of anterior horn (AH) in lumbar spinal cord (LSC) sections of 5-month-old WT and TDP-∆C (∆C) mice stained with anti-TDP-43(3H8) or anti-FLAG antibody. Scale bars: 20 μm. **d** Subcellular fractionation of LSC and cerebral cortex from 5-months-old TDP-∆C mice. Immunoblotting analyses of cytosolic and nuclear fractions from the indicated tissues of TDP-∆C mice using antibodies for TDP-43(3H8), FLAG, fibrillarin, and heat shock protein 110 (Hsp110). Note that endogenous TDP-WT and TDP-∆C protein were predominantly localized in nucleus and cytosol, respectively. **e-g** Immunoblotting analyses of endogenous TDP-43 and TDP-∆C proteins in brain and whole spinal cords (SC) detected by anti-TDP-43 antibody recognizing the amino acids near Ala 260 (A260) of TDP-43 or anti-FLAG antibody (**e**). A filled arrowhead indicates endogenous murine TDP-43, and open arrowheads indicate TDP-∆C, respectively. An asterisk denotes a non-specific band. Quantification of TDP-∆C relative to endogenous TDP-43 (**f**) or endogenous TDP-43 levels normalized to GAPDH (**g**) are plotted. Note that reduction of TDP-∆C protein levels was observed in both spinal cords and brains (**f**). While, the levels of endogenous TDP-43 protein were not affected by TDP-∆C (**g**). **h** and **i** Cycloheximide (CHX) chase assay revealed that TDP-∆C protein is less stable than TDP-WT protein. Mouse neuroblastoma Neuro2a (N2a) cells were transfected with expression plasmids for 3 × FLAG-tagged wild-type human TDP-43 (TDP-43(WT)-FLAG) or 3 × FLAG-tagged human TDP-43 mutant devoid of its C-terminal domain (TDP-∆C), and treated with CHX (15 μg/mL) for the indicated times. Cell lysates were then prepared and subjected to immunoblotting. Representative immunoblots using anti-FLAG and anti-β-actin antibodies are shown (**h**). Quantification of TDP-43(WT)-FLAG and TDP-∆C immunoblots relative to 0 h are plotted as mean ± standard error of the mean (SEM) (*n* = 3) Indicated *p*-values are results of multiple comparison *t*-test between TDP-43(WT) and TDP-∆C at the same time points (**i**)

In TDP-∆C mice, the expression pattern of TDP-ΔC proteins across various tissues including the central nervous system was similar to the one of wild-type TDP-43 protein (Fig. 2a, b). To assess the subcellular localization of TDP-ΔC protein in the mouse spinal cord, we immunostained lumbar spinal cord sections using an antibody against the endogenous TDP-43 (anti-TDP-43 (3H8), a mouse monoclonal antibody recognizing the TDP-43 C-terminal region) or an anti-FLAG antibody specifically recognizing (FLAG-tagged) TDP-ΔC protein (Fig. 2a, Additional file 1: Figure S2). TDP-ΔC protein, visualized by the anti-FLAG antibody, was localized both in the cytosol and nucleus of spinal motor neurons (Fig. 2c, upper panels), and did not affect the localization of endogenous wild-type TDP-43 (Fig. 2c, lower panels). Subcellular fractionation of TDP-ΔC mouse spinal cords revealed that TDP-ΔC protein was predominantly localized in cytosol (Fig. 2d). Although the mRNA level of TDP-ΔC was almost the same as endogenous TDP-43 mRNA (Fig. 1c), the steady-state level of TDP-∆C protein was much less than that of endogenous TDP-43 (Fig. 2e, f). While, the level of endogenous TDP-43 was not altered between TDP-∆C mice and controls (Fig. 2e, g). These results suggest that TDP-∆C protein is much less stable than the wild-type TDP-43. Consistent with this observation, TDP-ΔC protein was degraded apparently faster than TDP-43 protein in mouse neuroblastoma Neuro2a cells as evidenced by cycloheximide (CHX) chase assay (Fig. 2h, i).

### TDP-ΔC mice show age-dependent mild motor dysfunction

**Fig. 3:**
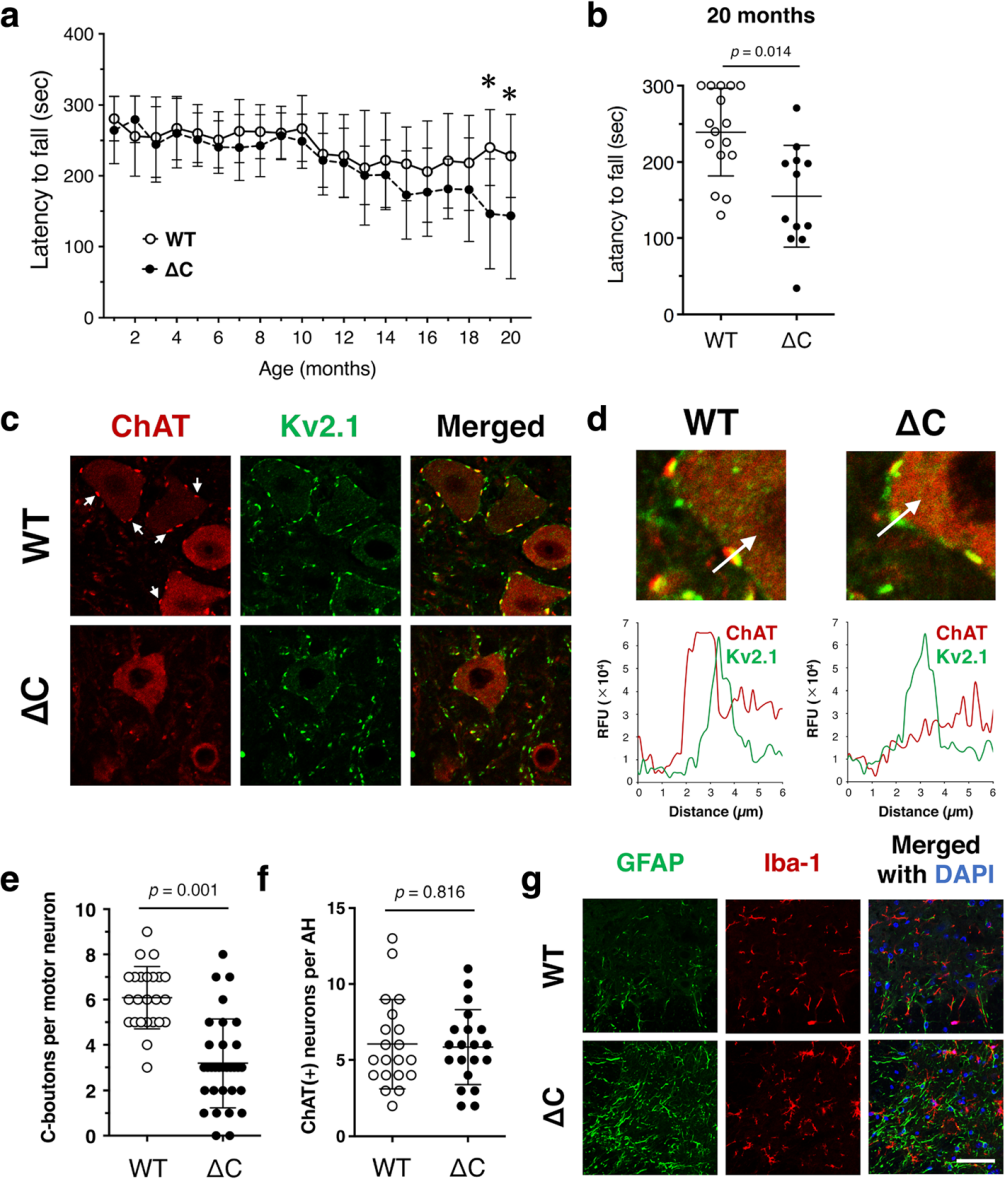
TDP-∆C knock-in mice show mild age-dependent motor dysfunction. **a** and **b** Rotarod performances of wild-type mice (WT, *n* = 16 (male: 11, female: 5)) and TDP-∆C mice (∆C, *n* = 12 (male: 5, female: 7)) were evaluated every month. Mean holding times on a rotating rod at the indicated ages are plotted with SD. The rotarod performance of TDP-∆C mice did not differ from WT until 18 months of age (**a**), but declined substantially at 19 and 20 months old (**b**). Asterisks represents *p* < 0.05. **c** and **d** The number of presynaptic terminals of C-boutons (C, arrows), represented by choline acetyl transferase (ChAT)-positive puncta surrounding motor neurons, was specifically reduced in 700-day-old TDP-∆C mice (**c**). Representative immunofluorescence images of lumbar motor neurons with C-boutons in 700-day-old WT and TDP-∆C mice stained with antibodies for ChAT and Kv2.1 along with the merged images. Loss of ChAT immunoreactivity on the C-boutons was also confirmed by fluorescent intensity profile (**d**). Scale bars: 20 μm. **e** and **f** The numbers of intact C-boutons per motor neuron (**e**) and motor neurons per each anterior horn (AH) (**f**) in lumbar spinal cord (LSC) of 700-day-old WT and TDP-∆C mice with indicated genotypes are shown. For quantification, more than 50 motor neurons (**e**) and 30 AHs (**f**) in three animals per genotype were counted, and the data are plotted as mean ± SD. **g** Gliosis of glial fibrillary acidic protein (GFAP)-positive astrocytes was slightly more intense in the LSC of 700-day-old TDP-∆C mice compared to age-matched WT. Representative immunofluorescence images of LSC sections from WT and TDP-∆C mice stained using anti-Iba1 (microglial marker, red) and anti-GFAP (astrocytic marker, green) antibodies, along with merged images. Sections were also counterstained with DAPI staining (blue). Scale bars: 20 μm

To examine whether there were any motor phenotypes in TDP-ΔC mice, we performed a rotarod test monthly from 2 to 20 months of age (Fig. 3a). Although no difference between genotypes was detected until 18 months old, a mild but significant decline in the rotarod performance in TDP-ΔC mice was observed both at 19 and 20 months old (Fig. 3a and b). This decline was observed in both genders (Additional file 1: Figure S3). As a first step to reveal the mechanistic basis for motor dysfunction in aged TDP-ΔC mice, we examined the C-boutons, large cholinergic synapses terminated on α-motor neurons, by immunofluorescence staining for staining for the presynaptic marker choline acetyltransferase (ChAT) and the post-synaptic marker Kv2.1 [15, 21, 49]. According to ChAT immunostaining, the number of C-boutons was substantially reduced in the lumbar spinal cord of aged TDP-∆C mice compared to age-matched WTs (Fig. 3c-e). Intriguingly, while ChAT-positive presynaptic boutons were reduced in number, whereas Kv2.1 labeled postsynaptic density seemed to be unchanged (Fig. 3d). Moreover, the number of ChAT-positive motor neurons was unaffected (Fig. 3f), indicating that there was no detectable loss of spinal motor neurons. We also analyzed neuromuscular junctions (NMJs) of tibialis anterior muscle in aged TDP-∆C mice, however, NMJs were preserved in 700-days-old TDP-∆C mice (Additional file 1: Figure S4). Inflammatory responses of microglia were not detectable, however, slight activation of astrocytes was observed in aged TDP-∆C mice as evidenced by increased expression of glial fibrillary acidic protein (GFAP) (Fig. 3g). This finding may reflect the astrocytic response to the synaptic disruption of C-boutons.

### Intranuclear TDP-∆C provokes cytotoxicity in cultured neuronal cells

**Fig. 4:**
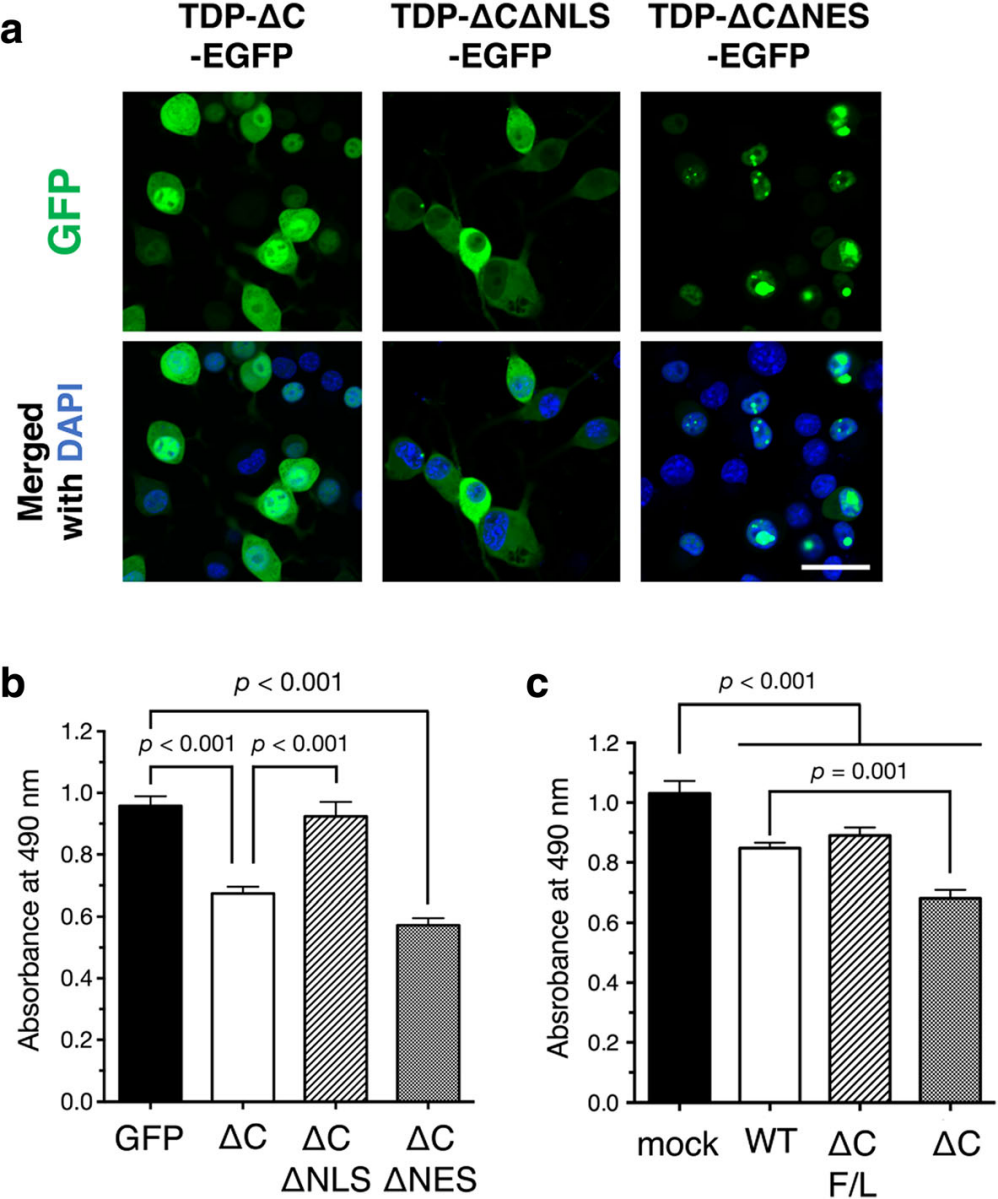
Intranuclear TDP-∆C induces cytotoxicity of cultured neuronal cells. **a** Representative images show subcellular localization of C-terminal EGFP-tagged TDP-∆C (TDP-∆C-EGFP), TDP-∆C with mutations in the NLS (TDP-∆C∆NLS-EGFP), and TDP-∆C with mutations in the NES (TDP-∆C∆NES-EGFP) expressed in mouse neuroblastoma Neuro2a (N2a) cells as well as merged images with DAPI. Scale bar: 20 μm. **b** and **c** Nuclear localization (**b**) and nucleotide binding ability (**c**) of TDP-∆C are crucial for cytotoxicity. N2a cells were transfected with the indicated expression plasmids and incubated for 48 h in the differentiation media. Cell viability was measured by MTS assay. ∆C F/L represents a TDP-∆C variant with F147 L / F149 L mutations defective in nucleotide binding. Data are expressed as mean ± SEM of three independent experiments, each performed in triplicate

We then examined whether the aberrant subcellular localization of TDP-∆C is involved in the synaptic loss observed in aged TDP-∆C mice. In TDP-∆C protein, both nuclear localization signal (NLS) and nuclear export signal (NES) are retained, which may be responsible for their subcellular localization in each compartment (Figs. 2a, 4a). In cell viability assays using Neuro2a cells, a NES-deficient TDP-∆C variant (TDP-∆C∆NES) was predominantly localized in nucleus (Fig. 4a, right) and exhibited cytotoxicity at the similar level to TDP-∆C, whereas a NLS-deficient TDP-∆C variant (TDP-∆C∆NLS; Fig. 4a middle) was predominantly localized in cytosol and demonstrated no cytotoxicity (Fig. 4b). Moreover, another TDP-∆C variant carrying F148 L / F149 L mutations in the RRM1 domain (TDP-∆C(F/L)), which is nearly devoid of its nucleotide binding ability, showed less cytotoxicity than TDP-∆C (Fig. 4c). These results suggest that nucleotide binding ability and nuclear localization of TDP-∆C are crucial for the age-dependent motor dysfunction in TDP-∆C mice.

### TDP-ΔC induces widespread perturbation of gene expression in mice

**Fig. 5:**
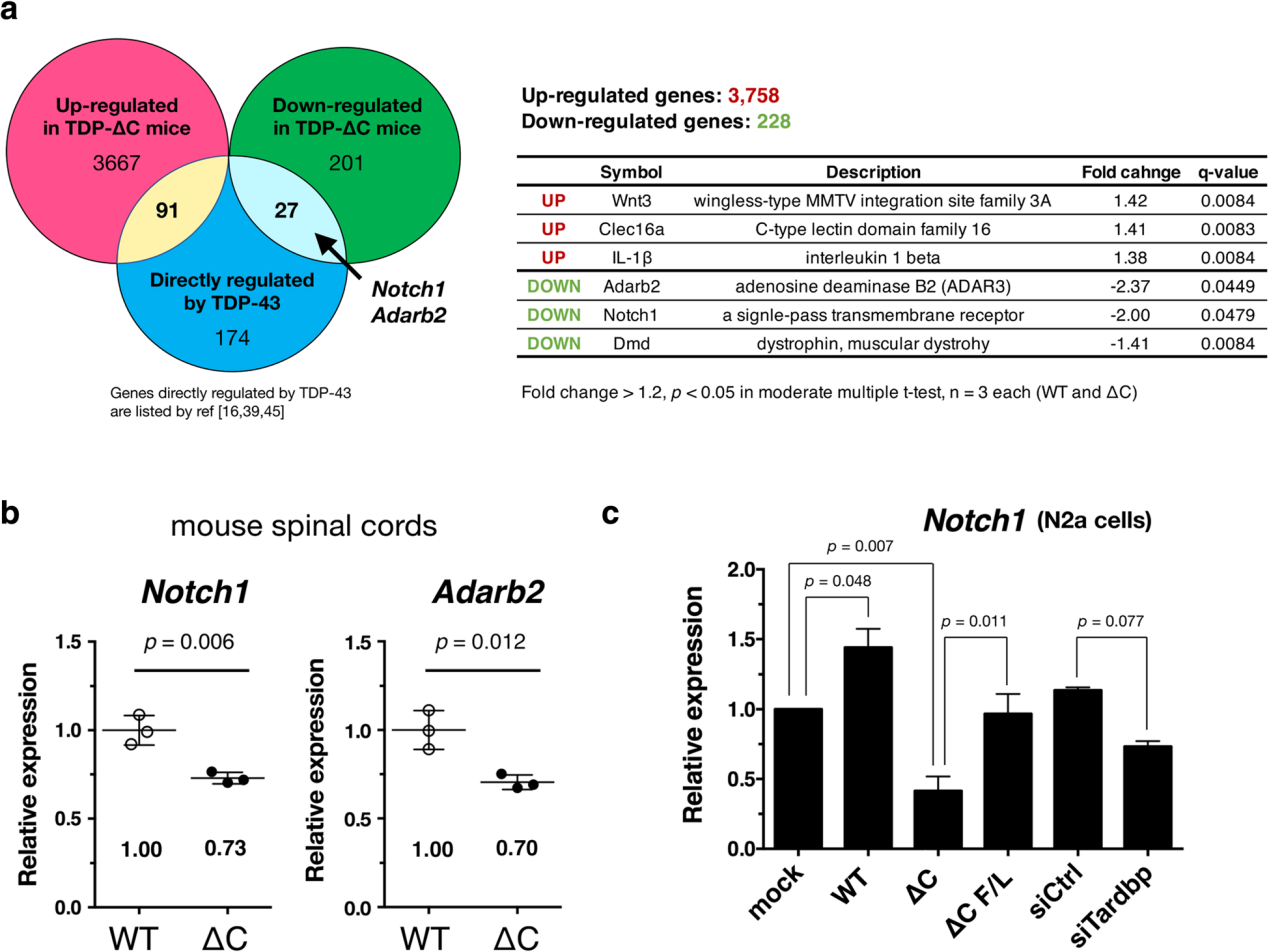
Gene expression levels are perturbed in spinal cord of aged TDP-∆C mice. **a** Venn diagram show deregulated genes in spinal cord (SC) of 700-day-old TDP-∆C mice identified by microarray analyses overlapped with ones directly regulated by TDP-43 (left). Representative deregulated genes, which are directly regulated by TDP-43 and related to motor dysfunction, in SC of 700-day-old TDP-∆C mice identified by microarray analyses (right). **b** Quantitative RT-PCR confirmed that levels of *Notch1* and *Adarb2* mRNA were downregulated in SC of 700-day-old TDP-∆C mice. The data are plotted as mean ± SD. **c** TDP-∆C reduced *Notch1* mRNA levels in Neuro2a (N2a) cells. N2a cells were transfected with the indicated expression plasmids or siRNAs. After 24 h of the transfection, total RNA was isolated, and the *Notch1* mRNA levels were quantified using quantitative RT-PCR. Note that both TDP-∆C overexpression (∆C) and *Tardbp* siRNA treatment (siTardbp) suppressed the *Notch1* mRNA levels. ∆C F/L represents the TDP-∆C mutant lacking the nucleotide binding ability as indicated in Fig. 4c. The data are plotted as mean ± SEM (*n* = 3)

To identify key genes linked to the motor dysfunction in TDP-∆C mice, we performed a microarray analysis using RNAs isolated from the aged TDP-∆C mouse spinal cords. We detected around 4,000 genes, of which 3,758 were upregulated (Additional file 2: Table S1) and 228 were downregulated (Additional file 3: Table S2) with fold-change > 1.2 (*q-value* < 0.05 in moderate t-test) (Fig. 5a). Among these differentially expressed genes, 118 genes (91 upregulated and 27 downregulated genes) are known to be directly regulated by TDP-43 (Additional file 4: Table S3) according to the data on RNA targets of TDP-43 in primary neurons [6, 30, 45]. Representative genes from this target group are listed in Fig. 5a. Especially among these affected genes, we confirmed downregulation of *Notch1* and *Adarb2* in TDP-∆C mice using quantitative RT-PCR (Fig. 5b). To determine the role of TDP-∆C in downregulation of *Notch1* mRNA, we measured the mRNA level of *Notch1* in Neuro2a cells with TDP-43 depletion or TDP-∆C overexpression. Intriguingly, downregulation of *Notch1* was observed in both the conditions, whereas overexpression of wild-type TDP-43 elevated the level of *Notch1* mRNA (Fig. 5c).

### Downregulation of Notch1 is associated with suppression of Akt signaling pathway in the aged TDP-∆C mice

To reveal the pathomechanism common to TDP-∆C mice and sporadic ALS, we focused on 118 genes known to be regulated by TDP-43 among the 4,000 differentially expressed genes in TDP-∆C mice (Fig. 5a). Since TDP-43 pathology is observed in motor neurons of almost all patients with sporadic ALS, the genes directly regulated by TDP-43 are of great potential importance to understand the mechanisms for motor dysfunction and synaptic abnormality observed in TDP-∆C mice, and may be relevant to pathogenic mechanisms for sporadic ALS.

**Fig. 6:**
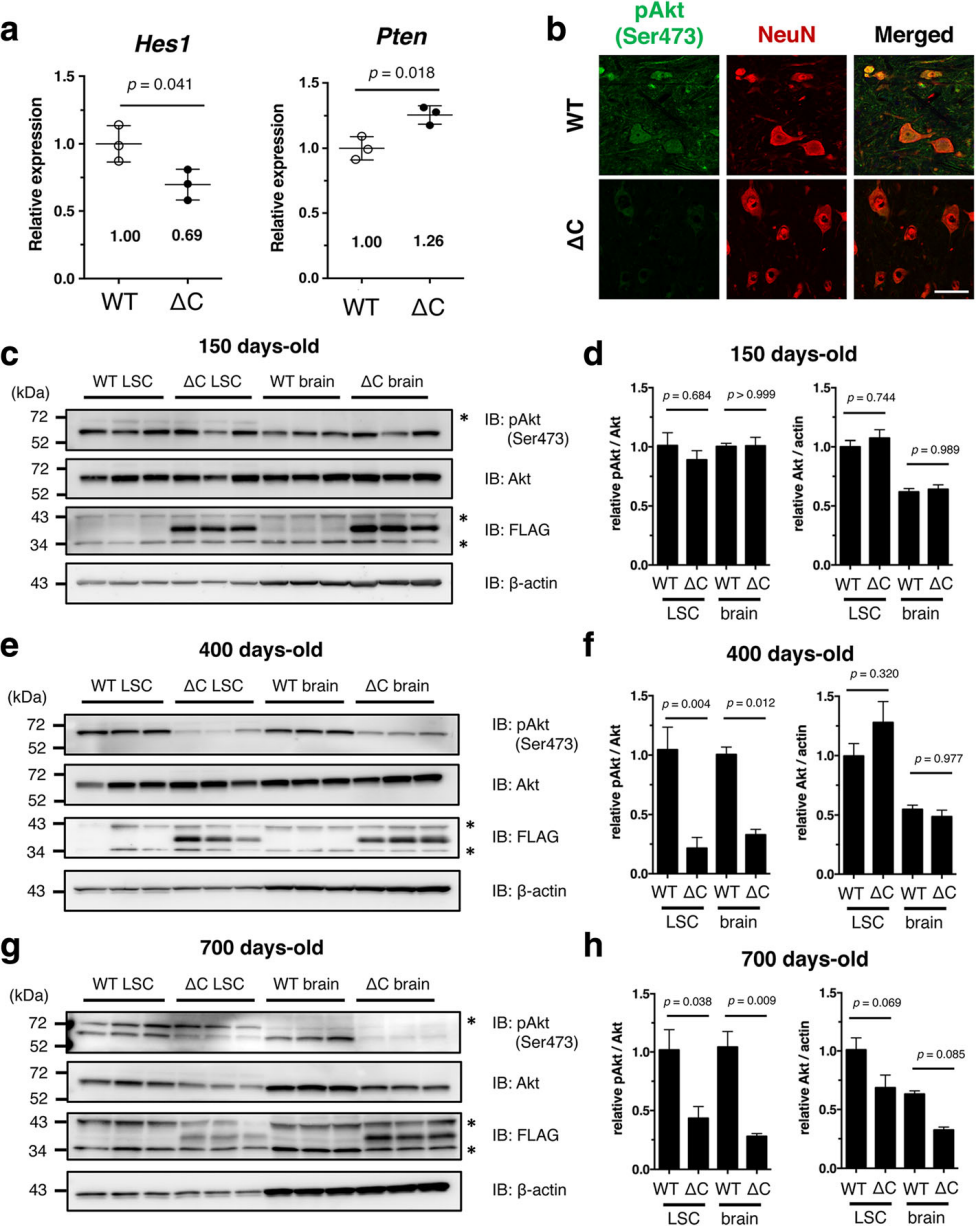
Age-dependent suppression of the Akt survival pathway in brain and spinal cord of TDP-∆C mice. **a** Quantitative RT-PCR confirmed downregulation of *Hes1* and upregulation of *Pten* in spinal cord of 700-day-old TDP-∆C (∆C) mice as compared to age-matched wild-type (WT) control mice. The mRNA levels relative to WT are shown as mean ± SD. **b** Representative images showing the lumbar spinal cord (LSC) anterior horn (AH) of WT and TDP-∆C mice at 700 days of age stained with antibodies for NeuN (red) and (active) phosphorylated-Akt (pAkt) (green) along with the merged images. Scale bar: 50 μm. **c-h** Progressive loss of active pAkt was observed in LSC and brain of TDP-∆C mice. Nervous tissues of WT or TDP-∆C mice at the indicated ages were analyzed by immunoblotting (**c**, **e**, **g**). Band intensities were semi-quantified using β-actin as an internal control and the relative ratios of pAkt/Akt (left panel) and Akt/Actin (right panel) are plotted (**d**, **f**, **h**). Asterisks are non-specific bands. The data are shown as mean ± SEM (*n* = 3)

**Fig. 7:**
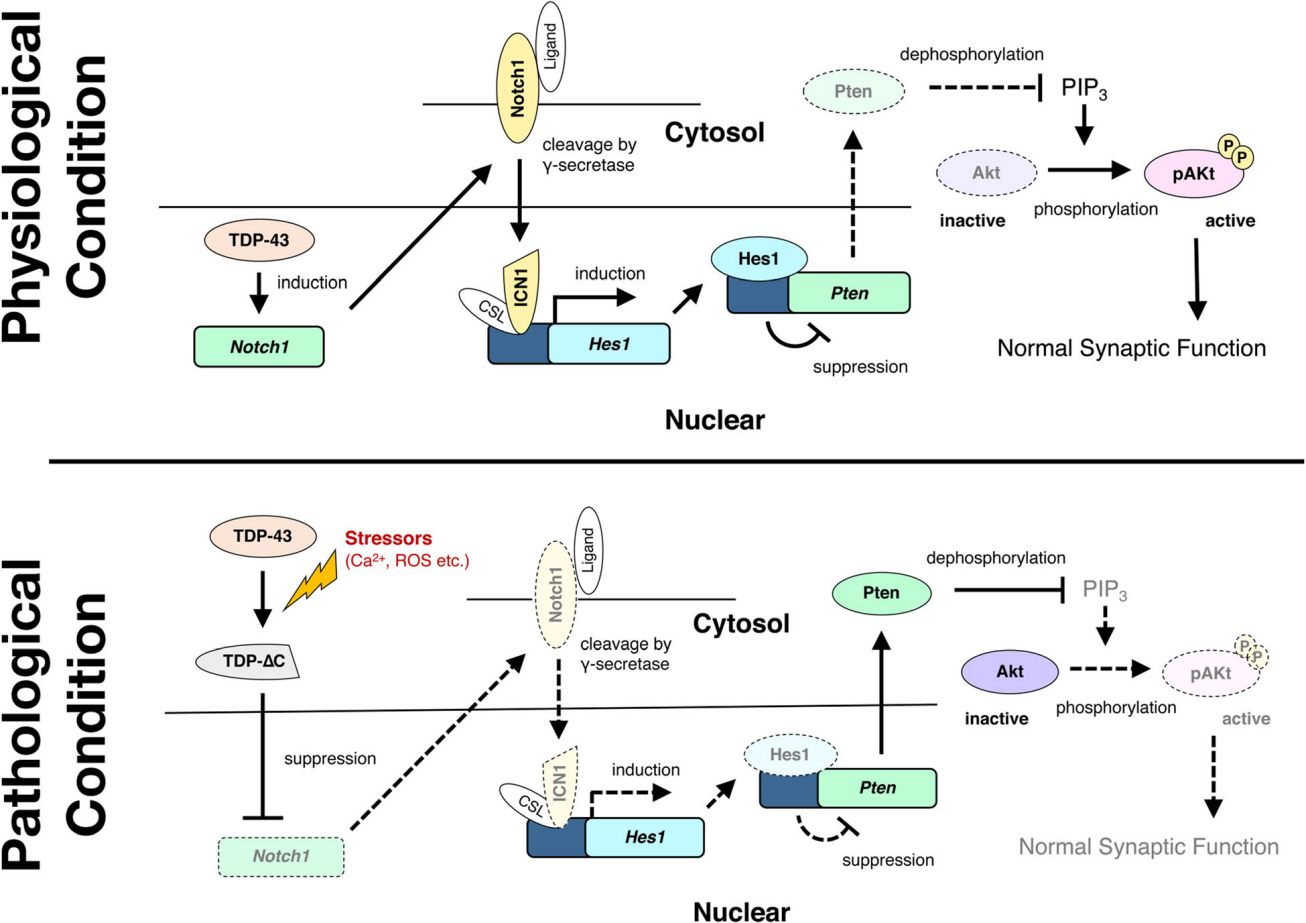
Schematic representation of *Notch1*-mediated regulation of Akt signaling disturbed by TDP-∆C protein. *Notch1* is transcriptionally regulated by TDP-43 protein (Fig. 5). Under physiological conditions, *Notch1* indirectly suppresses *Pten* expression, a negative regulator of Akt signaling, through transcriptional regulation of *Hes1* (upper panel). Under pathological conditions, however, the cleaved TDP-43 N-terminal fragment (TDP-∆C) reduces *Notch1* expression. Decreased *Notch1* results in increased *Pten* expression leading to the inactivation of Akt (lower panel). Inactivation of Akt may disturb normal synaptic function of motor neurons and cause motor dysfunction

Of these 118 genes, we focused on the Notch1-mediated regulation of Akt/PKB signaling, since alternation of *Notch1* has been reported in some ALS models or patients [17, 41, 48], and Akt is tightly involved in the maintenance of synaptic integrity and motor neuron survival [9, 23, 40, 52]. Molecular pathways linking *Notch1* and Akt have been demonstrated in previous studies [13, 50] as illustrated in Fig. 7. Briefly, *Notch1* increases transcription of its downstream target *Hes1*, and expression of *Pten*, a suppressor of phosphoinositide-3-dependent Akt activation, is downregulated by *Hes1* induction. Through this pathway, therefore, *Notch1* positively regulates Akt activity to maintain the synaptic integrity. We hypothesized that reduced expression of *Notch1* suppresses Akt activity, thereby disrupting synaptic structure in the aged TDP-∆C mice. As expected, the level of *Hes1* mRNA was reduced to about 69% of WT, and *Pten* mRNA was reversely increased to 126% of WT in aged TDP-∆C mice (Fig. 6a). The expression of phosphorylated Akt (pAkt), an active form of Akt, was substantially diminished in the spinal cord of the aged TDP-∆C mouse (Fig. 6b). Moreover, we found that the level of pAkt was reduced in an age-dependent manner. In young TDP-∆C mice (150 days-old), the level of pAkt did not differ from the WTs (Fig. 6c and d). However, a significant reduction of pAkt was observed in 400-day-old (Fig. 6e and f) and 700-day-old TDP-∆C mice (Fig. 6g and h). Total amounts of Akt proteins were also decreased in 700-day-old TDP-∆C mice compared to age-matched WTs, suggesting that chronic inactivation of Akt promotes degradation of Akt itself likely through a mechanism analogous to that described previously in damaged neurons [38].

## Discussion

In this study, we established TDP-∆C knock-in mice, and demonstrated age-dependent motor dysfunction associated with a substantial loss of C-boutons on lumbar motor neurons. TDP-∆C deregulated numerous genes, and suppression of the Notch1-Akt signaling pathway was associated with structural abnormalities of C-boutons. These data suggest that TDP-∆C contributes to the motor dysfunction associated with impaired Notch- Akt signaling pathway (Fig. 7).

Consistent with a previous study [4], TDP-∆C leaked from nucleus to cytosol. However, the mechanism for this nucleocytoplasmic translocation of TDP-43 is controversial. One study suggested that TDP-43 translocation from nucleus to cytosol is mediated by the exportin family [3], whereas recent studies suggested that nuclear export of TDP-43 is dependent on diffusion rather than the predicted NES [10, 26]. In the present study, GFP-fused TDP-∆C, which molecular size (~ 70 kDa) is much larger than the average size of nuclear pore (~ 40 kDa), leaked from nuclei as well as FLAG-tagged TDP-∆C protein, while the TDP-∆C∆NES was retained, suggesting that the leakage of TDP-∆C is likely to be dependent on active nuclear export machinery (possibly mediated by an exportin family). Since nuclear-localized TDP-∆C exhibited cytotoxicity, clearance of TDP-43 from nuclei in ALS may be a result of cellular stress response. Future studies are required to investigate the detailed mechanisms of TDP-43 translocation.

Previous studies including ours reported a reduced number of C-boutons on motor neurons in both ALS model mice expressing mutant SOD1 [12, 15, 18] and patients with ALS [15, 22]. Since cholinergic interneurons control the firing frequency of α-motor neurons through C-boutons [7], a loss of C-boutons may induce hyperexcitation of α-motor neurons leading to motor dysfunction and eventual neurodegeneration. The number of C-boutons was substantially reduced in aged TDP-∆C mice while there was no detectable loss of spinal α-motor neurons. Considering that a reduction of C-boutons was observed prior to the disease onset in the studies using SOD1 mice [12, 15, 18], TDP-∆C-mediated neurotoxicity may not be sufficient to induce a substantial loss of α-motor neurons within the two-year lifespan of mice. Furthermore, NMJs of TDP-∆C mice were relatively preserved, suggesting the possibility that dysfunction of C-boutons is the earliest neuropathological phenotype in motor dysfunction related to TDP-43. The molecular basis for this selective loss of C-boutons in aged TDP-∆C mice requires further investigation.

TDP-∆C perturbed the expression levels of around 4,000 genes in mouse spinal cords; unexpectedly, however, a limited number of these genes are regulated by TDP-43 according to the studies screening for its neuronal RNA targets [6, 30, 45]. While on the other hand, nuclear-localized TDP-∆C was sufficient to induce cytotoxicity. These findings suggest that TDP-∆C evokes neurotoxicity not simply by inhibiting the functions of TDP-43. More importantly, our results suggest that TDP-43 N-terminal fragments are not merely innocuous byproducts of cleaved TDP-43 proteins, but are key elements to induce neurotoxicity. Intriguingly, mild motor dysfunction similar to the one observed in TDP-∆C mice was also observed in *Tardbp* heterozygous knock-out mice generated by a gene-trap insertion strategy [14] or *Tardbp*^Q101X^ heterozygous knock-in mice generated by an N-ethyl -N-nitrosourea (ENU) induced mutagenesis [28]. In these mice, as similar to TDP-∆C mice, the N-terminal fragments of TDP-43 were additionally expressed without affecting the levels of endogenous WT TDP-43, supporting the notion that the N-terminal fragments of TDP-43 are involved in age-dependent motor dysfunction in mice. Although many previous studies have identified the C-terminal fragment of TDP-43 as a core component of TDP-43 protein aggregates, recent studies have revealed that the TDP-43 N-terminal region promotes dimerization of TDP-43 protein [1, 29, 39]. Further, our studies and those of others have strongly implicated TDP-43 N-terminal fragments in ALS pathogenesis [31, 46].

Among deregulated genes in aged TDP-∆C mouse spinal cord, we identified downregulation of Notch1 − Akt signaling genes during age-dependent motor dysfunction. Moreover, we found that the levels of pAkt and total Akt gradually decreased with age in the nervous tissue of TDP-∆C mice. Although direct evidence for the link between chronic downregulation of pAkt and age-dependent motor dysfunction in TDP-∆C mice was not provided in this study, there are several studies suggesting the potential role of impaired Akt signaling in ALS pathomechanisms. Indeed, reduced Notch1 signaling was also observed in a C9orf72-linked ALS model [48]. The Pten−Akt axis was disrupted by the C9orf72-related (G4C2) RNA repeat, and partial depletion of *Pten* ameliorated the repeat-mediated toxicity [32]. Moreover, reduced Akt signaling was also reported in SOD1-related ALS models [8, 42]. We found that expression of the Notch1 was also reduced by siRNA-mediated depletion of TDP-43 in cultured neuronal cells, a finding potentially relevant to sporadic ALS characterized by a loss of nuclear TDP-43. Although one study showed that TDP-43 or mutant SOD1 overexpression resulted in neurodegeneration through hyperactive Notch1 signaling [41], most of studies cited here are consistent with our findings, suggesting that insufficient Notch1-Akt signaling may lead to neurotoxicity and motor neuron dysfunction in ALS.

## Conclusions

Gene ablation of the TDP-43 C-terminal domain in mice (TDP-∆C mice) induced age-dependent motor dysfunction associated with loss of cholinergic synapses on spinal α-motor neurons. This age-dependent motor impairment was also associated with suppression of Notch1-Akt signaling pathway. Our data uncovered a detrimental role of N-terminal TDP-43 fragments in ALS pathology in mice, associated with suppression of Akt surviving signal.

## Additional files

Additional file 1: **Figure S1.** Complete deletion of C-terminal domain from TDP-43 induced severe neurotoxicity in cultured neuronal cells. **Figure S2.** Anti-TDP-43(3H8) recognizes C-terminal domain of TDP-43. **Figure S3.** No gender differences were found in rotarod scores in aged WT and TDP-∆C mice. **Figure S4.** Neuro-muscular junction (NMJ) was not affected in aged TDP-∆C mice. (PDF 893 kb)

Additional file 2: **Table S1.** Genes upregulated (> 1.2 fold) in 700-day-old TDP-∆C mouse spinal cord. (XLSX 499 kb)

Additional file 3: **Table S2.** Genes downregulated (> − 1.2 fold) in 700-day-old TDP-∆C mouse spinal cord. (XLSX 41 kb)

Additional file 4: **Table S3.** Affected genes (> 1.2 or < − 1.2 fold) among known TDP-43 regulatory targets in 700-day-old TDP-∆C mouse spinal cord. (XLSX 20 kb)

## Data Availability

Data, material and software information supporting the conclusions of this article are included within the article and its additional files.

## Abbreviations

ALS: Amyotrophic lateral sclerosis
FTLD: Frontotemporal lobar degeneration
NES: Nuclear export signal
NLS: Nuclear localization signal
SOD1: Cu/Zn superoxide dismutase
TDP-43: TAR DNA-binding protein 43

## References

[1] Afroz T, Hock EM, Ernst P, Foglieni C, Jambeau M, Gilhespy LAB, Laferriere F, Maniecka Z, Pluckthun A, Mittl P (2017). Functional and dynamic polymerization of the ALS-linked protein TDP-43 antagonizes its pathologic aggregation. Nat Commun.

[2] Arai T, Hasegawa M, Akiyama H, Ikeda K, Nonaka T, Mori H, Mann D, Tsuchiya K, Yoshida M, Hashizume Y (2006). TDP-43 is a component of ubiquitin-positive tau-negative inclusions in frontotemporal lobar degeneration and amyotrophic lateral sclerosis. Biochem Biophys Res Commun.

[3] Archbold HC, Jackson KL, Arora A, Weskamp K, Tank EM, Li X, Miguez R, Dayton RD, Tamir S, Klein RL (2018). TDP43 nuclear export and neurodegeneration in models of amyotrophic lateral sclerosis and frontotemporal dementia. Sci Rep.

[4] Ayala YM, Zago P, D'Ambrogio A, Xu YF, Petrucelli L, Buratti E, Baralle FE (2008). Structural determinants of the cellular localization and shuttling of TDP-43. J Cell Sci.

[5] Chen-Plotkin AS, Lee VM, Trojanowski JQ (2010). TAR DNA-binding protein 43 in neurodegenerative disease. Nat Rev Neurol.

[6] Colombrita C, Onesto E, Megiorni F, Pizzuti A, Baralle FE, Buratti E, Silani V, Ratti A (2012). TDP-43 and FUS RNA-binding proteins bind distinct sets of cytoplasmic messenger RNAs and differently regulate their post-transcriptional fate in motoneuron-like cells. J Biol Chem.

[7] Deardorff AS, Romer SH, Sonner PM, Fyffe RE (2014) Swimming against the tide: investigations of the C-Bouton synapse. Front Neural Circuits 8: 106 Doi 10.3389/fncir.2014.00106 10.3389/fncir.2014.00106 PMC4167003 25278842

[8] Dewil M, Lambrechts D, Sciot R, Shaw PJ, Ince PG, Robberecht W, Van den Bosch L (2007). Vascular endothelial growth factor counteracts the loss of phospho-Akt preceding motor neurone degeneration in amyotrophic lateral sclerosis. Neuropathol Appl Neurobiol.

[9] Di Polo A (2015). Dendrite pathology and neurodegeneration: focus on mTOR. Neural Regen Res.

[10] Ederle H, Funk C, Abou-Ajram C, Hutten S, Funk EBE, Kehlenbach RH, Bailer SM, Dormann D (2018). Nuclear egress of TDP-43 and FUS occurs independently of Exportin-1/CRM1. Sci Rep.

[11] Furukawa Y, Kaneko K, Watanabe S, Yamanaka K, Nukina N (2011). A seeding reaction recapitulates intracellular formation of Sarkosyl-insoluble transactivation response element (TAR) DNA-binding protein-43 inclusions. J Biol Chem.

[12] Gallart-Palau Xavier, Tarabal Olga, Casanovas Anna, Sábado Javier, Correa Francisco J., Hereu Marta, Piedrafita Lídia, Calderó Jordi, Esquerda Josep E. (2014). Neuregulin-1 is concentrated in the postsynaptic subsurface cistern of C-bouton inputs to α-motoneurons and altered during motoneuron diseases. The FASEB Journal.

[13] Hales EC, Taub JW, Matherly LH (2014). New insights into Notch1 regulation of the PI3K-AKT-mTOR1 signaling axis: targeted therapy of gamma-secretase inhibitor resistant T-cell acute lymphoblastic leukemia. Cell Signal.

[14] Kraemer BC, Schuck T, Wheeler JM, Robinson LC, Trojanowski JQ, Lee VM, Schellenberg GD (2010). Loss of murine TDP-43 disrupts motor function and plays an essential role in embryogenesis. Acta Neuropathol.

[15] Lasiene J, Komine O, Fujimori-Tonou N, Powers B, Endo F, Watanabe S, Shijie J, Ravits J, Horner P, Misawa Het al (2016) Neuregulin 1 confers neuroprotection in SOD1-linked amyotrophic lateral sclerosis mice via restoration of C-boutons of spinal motor neurons. Acta Neuropathol Commun 4: 15 Doi 10.1186/s40478-016-0286-7 10.1186/s40478-016-0286-7 PMC4758105 26891847

[16] Lattante S, Rouleau GA, Kabashi E (2013). TARDBP and FUS mutations associated with amyotrophic lateral sclerosis: summary and update. Hum Mutat.

[17] Ma X, Drannik A, Jiang F, Peterson R, Turnbull J (2017). Crosstalk between notch and sonic hedgehog signaling in a mouse model of amyotrophic lateral sclerosis. Neuroreport.

[18] Milan L, Courtand G, Cardoit L, Masmejean F, Barriere G, Cazalets JR, Garret M, Bertrand SS (2015). Age-related changes in pre- and postsynaptic Partners of the Cholinergic C-boutons in wild-type and SOD1G93A lumbar Motoneurons. PLoS One.

[19] Mishina M, Sakimura K (2007). Conditional gene targeting on the pure C57BL/6 genetic background. Neurosci Res.

[20] Motohashi K (2015). A simple and efficient seamless DNA cloning method using SLiCE from Escherichia coli laboratory strains and its application to SLiP site-directed mutagenesis. BMC Biotechnol.

[21] Muennich EA, Fyffe RE (2004). Focal aggregation of voltage-gated, Kv2.1 subunit-containing, potassium channels at synaptic sites in rat spinal motoneurones. J Physiol.

[22] Nagao M, Misawa H, Kato S, Hirai S (1998). Loss of cholinergic synapses on the spinal motor neurons of amyotrophic lateral sclerosis. J Neuropathol Exp Neurol.

[23] Namikawa K, Honma M, Abe K, Takeda M, Mansur K, Obata T, Miwa A, Okado H, Kiyama H (2000). Akt/protein kinase B prevents injury-induced motoneuron death and accelerates axonal regeneration. J Neurosci.

[24] Neumann M, Sampathu DM, Kwong LK, Truax AC, Micsenyi MC, Chou TT, Bruce J, Schuck T, Grossman M, Clark CM (2006). Ubiquitinated TDP-43 in frontotemporal lobar degeneration and amyotrophic lateral sclerosis. Science.

[25] Nonaka T, Kametani F, Arai T, Akiyama H, Hasegawa M (2009). Truncation and pathogenic mutations facilitate the formation of intracellular aggregates of TDP-43. Hum Mol Genet.

[26] Pinarbasi ES, Cagatay T, Fung HYJ, Li YC, Chook YM, Thomas PJ (2018). Active nuclear import and passive nuclear export are the primary determinants of TDP-43 localization. Sci Rep.

[27] Ratti A, Buratti E (2016). Physiological functions and pathobiology of TDP-43 and FUS/TLS proteins. J Neurochem.

[28] Ricketts T, McGoldrick P, Fratta P, de Oliveira HM, Kent R, Phatak V, Brandner S, Blanco G, Greensmith L, Acevedo-Arozena A (2014). A nonsense mutation in mouse Tardbp affects TDP43 alternative splicing activity and causes limb-clasping and body tone defects. PLoS One.

[29] Sasaguri H, Chew J, Xu YF, Gendron TF, Garrett A, Lee CW, Jansen-West K, Bauer PO, Perkerson EA, Tong J (2016). The extreme N-terminus of TDP-43 mediates the cytoplasmic aggregation of TDP-43 and associated toxicity in vivo. Brain Res.

[30] Sephton CF, Cenik C, Kucukural A, Dammer EB, Cenik B, Han Y, Dewey CM, Roth FP, Herz J, Peng J (2011). Identification of neuronal RNA targets of TDP-43-containing ribonucleoprotein complexes. J Biol Chem.

[31] Seyfried NT, Gozal YM, Dammer EB, Xia Q, Duong DM, Cheng D, Lah JJ, Levey AI, Peng J (2010). Multiplex SILAC analysis of a cellular TDP-43 proteinopathy model reveals protein inclusions associated with SUMOylation and diverse polyubiquitin chains. Mol Cell Proteomics.

[32] Stopford MJ, Higginbottom A, Hautbergue GM, Cooper-Knock J, Mulcahy PJ, De Vos KJ, Renton AE, Pliner H, Calvo A, Chio A (2017). C9ORF72 hexanucleotide repeat exerts toxicity in a stable, inducible motor neuronal cell model, which is rescued by partial depletion of Pten. Hum Mol Genet.

[33] Taylor JP, Brown RH, Cleveland DW (2016). Decoding ALS: from genes to mechanism. Nature.

[34] Tsuiji H, Iguchi Y, Furuya A, Kataoka A, Hatsuta H, Atsuta N, Tanaka F, Hashizume Y, Akatsu H, Murayama S (2013). Spliceosome integrity is defective in the motor neuron diseases ALS and SMA. EMBO Mol Med.

[35] Tsuji H, Arai T, Kametani F, Nonaka T, Yamashita M, Suzukake M, Hosokawa M, Yoshida M, Hatsuta H, Takao M (2012). Molecular analysis and biochemical classification of TDP-43 proteinopathy. Brain.

[36] Udan M, Baloh RH (2011). Implications of the prion-related Q/N domains in TDP-43 and FUS. Prion.

[37] van Es MA, Hardiman O, Chio A, Al-Chalabi A, Pasterkamp RJ, Veldink JH, van den Berg LH (2017). Amyotrophic lateral sclerosis. Lancet.

[38] Wakatsuki S, Saitoh F, Araki T (2011). ZNRF1 promotes Wallerian degeneration by degrading AKT to induce GSK3B-dependent CRMP2 phosphorylation. Nat Cell Biol.

[39] Wang Ailin, Conicella Alexander E, Schmidt Hermann Broder, Martin Erik W, Rhoads Shannon N, Reeb Ashley N, Nourse Amanda, Ramirez Montero Daniel, Ryan Veronica H, Rohatgi Rajat, Shewmaker Frank, Naik Mandar T, Mittag Tanja, Ayala Yuna M, Fawzi Nicolas L (2018). A single N‐terminal phosphomimic disrupts TDP‐43 polymerization, phase separation, and RNA splicing. The EMBO Journal.

[40] Wang Q, Liu L, Pei L, Ju W, Ahmadian G, Lu J, Wang Y, Liu F, Wang YT (2003). Control of synaptic strength, a novel function of Akt. Neuron.

[41] Wang SY, Ren M, Jiang HZ, Wang J, Jiang HQ, Yin X, Qi Y, Wang XD, Dong GT, Wang TH (2015). Notch pathway is activated in cell culture and mouse models of mutant SOD1-related familial amyotrophic lateral sclerosis, with suppression of its activation as an additional mechanism of neuroprotection for lithium and valproate. Neuroscience.

[42] Warita H, Manabe Y, Murakami T, Shiro Y, Nagano I, Abe K (2001). Early decrease of survival signal-related proteins in spinal motor neurons of presymptomatic transgenic mice with a mutant SOD1 gene. Apoptosis.

[43] Watanabe S, Hayakawa T, Wakasugi K, Yamanaka K (2014). Cystatin C protects neuronal cells against mutant copper-zinc superoxide dismutase-mediated toxicity. Cell Death Dis.

[44] Watanabe S, Ilieva H, Tamada H, Nomura H, Komine O, Endo F, Jin S, Mancias P, Kiyama H, Yamanaka K (2016). Mitochondria-associated membrane collapse is a common pathomechanism in SIGMAR1- and SOD1-linked ALS. EMBO Mol Med.

[45] Xiao S, Sanelli T, Dib S, Sheps D, Findlater J, Bilbao J, Keith J, Zinman L, Rogaeva E, Robertson J (2011). RNA targets of TDP-43 identified by UV-CLIP are deregulated in ALS. Mol Cell Neurosci.

[46] Yamashita T, Hideyama T, Hachiga K, Teramoto S, Takano J, Iwata N, Saido TC, Kwak S (2012). A role for calpain-dependent cleavage of TDP-43 in amyotrophic lateral sclerosis pathology. Nat Commun.

[47] Yamashita T, Kwak S (2014). The molecular link between inefficient GluA2 Q/R site-RNA editing and TDP-43 pathology in motor neurons of sporadic amyotrophic lateral sclerosis patients. Brain Res.

[48] Yang D, Abdallah A, Li Z, Lu Y, Almeida S, Gao F-B (2015). FTD/ALS-associated poly (GR) protein impairs the notch pathway and is recruited by poly (GA) into cytoplasmic inclusions. Acta Neuropathol.

[49] Zagoraiou L, Akay T, Martin JF, Brownstone RM, Jessell TM, Miles GB (2009). A cluster of cholinergic premotor interneurons modulates mouse locomotor activity. Neuron.

[50] Zeng C, Xing R, Liu J, Xing F (2016). Role of CSL-dependent and independent notch signaling pathways in cell apoptosis. Apoptosis.

[51] Zhang Y, Werling U, Edelmann W (2012). SLiCE: a novel bacterial cell extract-based DNA cloning method. Nucleic Acids Res.

[52] Zhou H, Li XM, Meinkoth J, Pittman RN (2000). Akt regulates cell survival and apoptosis at a postmitochondrial level. J Cell Biol.

