# Supplementary material for: Mice deficient in the C-terminal domain of TAR DNA-binding protein 43 develop age-dependent motor dysfunction associated with impaired Notch1−Akt signaling pathway
Source: Acta Neuropathol Commun. 2019 Jul 25;7:118. doi: 10.1186/s40478-019-0776-5 (PMC6657153; doi:10.1186/s40478-019-0776-5)
Supplement: Supplementary file 1 — Figure S1. Complete deletion of C-terminal domain from TDP-43 induced severe neurotoxicity in cultured neuronal cells. Figure S2. Anti-TDP-43(3H8) recognizes C-terminal domain of TDP-43. Figure S3. No gender differences were found in rotarod scores in aged WT and TDP-∆C mice. Figure S4. Neuro-muscular junction (NMJ) was not affected in aged TDP-∆C mice. (PDF 893 kb) [file 40478_2019_776_MOESM1_ESM.pdf]

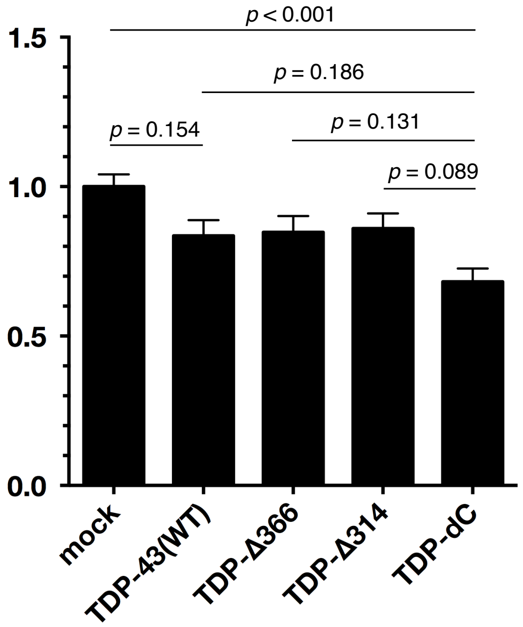

Figure S1. Complete deletion of C-terminal domain from TDP-43 induced severe neurotoxicity in cultured neuronal cells.

Neuro2a cells transfected with expression plasmids of 3xFLAG tagged human TDP-43 variants, and measured cell viability using MTS assay as described in *Materials & Methods* section. TDP-43(WT) represents full-length TDP-43. TDP-Δ366 or TDP-Δ314 represents a variant lacking C-terminal domain from a.a. 367-414 or a.a. 315-414, respectively. TDP-dC, in which the C-terminal domain (a.a. 275-414) was completely deleted, showed a trend of most severe neurotoxicity compared to the others. Data are shown as means ± SEM. *p*-values calculated by ANOVA following multiple comparison Tukey's *t*-test. n = 3, each experiment was performed in triplicate.

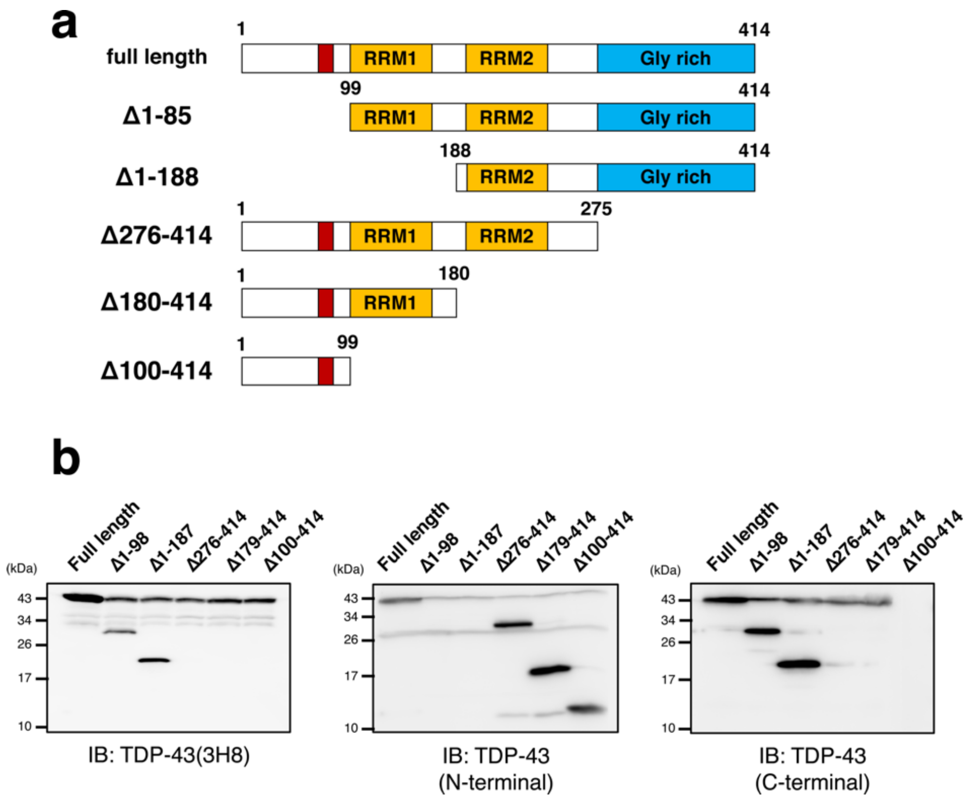

Figure S2. Anti-TDP-43(3H8) recognizes C-terminal domain of TDP-43

(A) Schematic illustration of human TDP-43 variants used for the antibody validation. (B) Representative immunoblotting image of the TDP-43 variants expressed in N2a cells. Note that both anti-TDP-43(3H8) and anti-TDP-43(C-terminal) recognized the TDP-43 variants devoid of its N-terminal domain, indicating that antigen of TDP-43(3H8) is the C-terminal domain (276-414) of TDP-43.

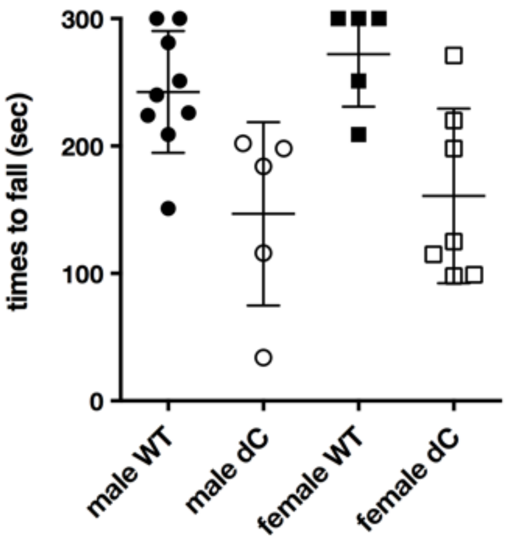

Figure S3. No gender differences were found in rotarod scores in aged WT and TDP-ΔC mice.

Rotarod scores of 20-month-old wild-type (WT) or TDP-ΔC (dC) mice, shown in Fig. 3b, in each gender were re-plotted. In both genders, dC mice showed lower rotarod score than WT. Difference between female WT and female dC was statistically significant (p = 0.025), and difference in male WT and male dC was a trend (p = 0.062). Moreover, no gender difference was observed in rotarod scores of both genotypes (p > 0.999 in both WT and ΔC). Statistical analyses were performed in Dunn's non-parametric multiple comparison test.

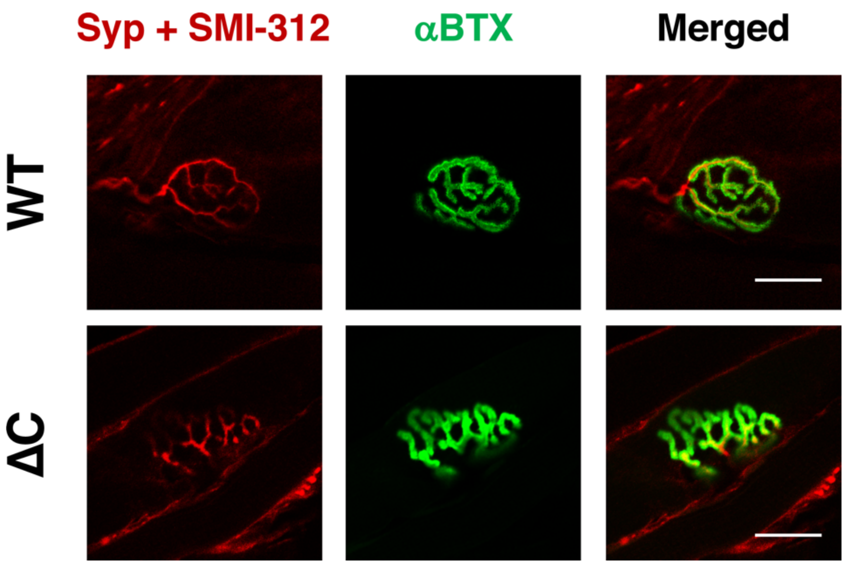

Figure S4. Neuromuscular junction (NMJ) was not affected in aged TDP-ΔC mice

NMJs in tibialis anterior muscle of 700 days-old wild-type (WT) or TDP-ΔC (ΔC) mouse were visualized by immunolabeling of synaptophysin (Syp) and SMI-312 (red), and Alexa488-conjugated α-bungarotoxin (αBTX), binding to acetylcholine receptor on muscles (green). No obvious denervation sign was observed in the aged TDP-ΔC mice. Scale bars: 20 µm.

Supplementary Experimental procedure

Validation of anti-TDP-43(N-terminal) and anti-TDP-43(3H8) antibody

cDNA of each human TDP-43 variants were sub-cloned into pF5K mammalian expression vector (Promega Biosciences, San Louis Obispo, CA). Neuro2a cells were transfected with Lipofectamine 2000 as manufacturer's instruction, and incubated for 24 h. The cell lysates (15 μg/lane) were prepared, and immunoblotted as described in the *Materials & Methods* section.

Immunofluorescence of NMJs

Tibialis anterior muscle of TDP-ΔC or wild-type mice fixed with 4 %(w/v) paraformaldehyde was embedded in Tissue-Tek OCT compound medium, and frozen at −80 ºC until use. After blocking, the 30 μm-sliced muscle sections were incubated with primary antibodies: anti-synaptophysin (1:1,000, #S5768, RRID:AB_477523, Sigma-Aldrich), anti-SMI-312 (1:1,000, # 837904, RRID: AB_2566782, Biolegend, San Diego, CA) for overnight at 4 ºC. Bound primary antibodies were detected with Alexa Fluor 546-conjugated anti-mouse IgG secondary antibody (both used in 1:1000) (Thermo Fisher Scientific Inc., Waltham, MA, USA). Acetylcholine receptors on the muscle were labeled with Alexa Fluor 488-conjugated α-bungarotoxin (1 μg/mL, Thermo). Immunofluorescence images were obtained by a confocal laser scanning microscopy (LSM-700; Carl Zeiss AG) and the equipped software (Zen; Carl Zeiss AG).
